# Robotic systems in total knee arthroplasty: current surgical trauma perspectives

**DOI:** 10.1093/burnst/tkac049

**Published:** 2022-12-16

**Authors:** Kai Lei, Li-Ming Liu, Lin Guo

**Affiliations:** Center for Joint Surgery, Southwest Hospital, Army Military Medical University, No. 30 Gaotanyan Street, Shapingba District, Chongqing, China, 400038; Center for Joint Surgery, Southwest Hospital, Army Military Medical University, No. 30 Gaotanyan Street, Shapingba District, Chongqing, China, 400038; Center for Joint Surgery, Southwest Hospital, Army Military Medical University, No. 30 Gaotanyan Street, Shapingba District, Chongqing, China, 400038

Total knee arthroplasty (TKA) has been a successful solution for end-stage osteoarthritis for decades. Restoring neutral lower limb alignment with a well-positioned prosthesis is considered one of the most important goals for successful TKA. Multiple new technologies, including robot-assisted TKA (RA-TKA), have been developed to achieve precise TKA surgery. Over decades of usage, robotic arm registration and installation have been approved for operative accuracy and criticized for increased perioperative times and surgical trauma. The limitation of surgical trauma is a rarely discussed but equally important factor when the decision to choose RA-TKA is about to be made.

The study “Robot-assisted surgery in total knee arthroplasty: trauma maker or trauma savior? A prospective, randomized cohort study by Xu *et al*. https://doi.org/10.1093/burnst/tkac034 [1] is a valuable attempt to improve the evaluation of perioperative surgical trauma. This study has added new knowledge to the current concept of surgical trauma in RA-TKA. We can learn from the results of monitoring perioperative inflammatory factors that RA-TKA might decrease, rather than increase, surgical trauma several days after surgery. The reasons could include that RA-TKA can shorten the time spent on bone cutting and gap balancing, which might compensate for the time consumed otherwise. Improved accuracy of mechanical alignment might decrease unnecessary soft tissue release, which decreases soft tissue trauma. The authors successfully discussed some key factors that could decrease intraoperative trauma, minimize the inflammatory response and favour postoperative comfort. However, there are more perioperative factors that can influence surgical trauma. If the decision to use RA-TKA is made, the surgeon should be aware of them. Herein, we review the existing viewpoints concerning the influence of RA-TKA surgical trauma to provide a broader range of vision for this subject.

## Factors that can decrease surgical trauma of RA-TKA

### Avoidance of unnecessary soft tissue release

The ascertained advantages of RA-TKA include planning precision, alignment accuracy and prosthetic outlier reduction. Decreased surgical trauma could be related to the avoidance of unnecessary soft tissue release, as reported by the authors of the article being commented on and by other researchers [2,3]. Soft tissue releases are performed in 50–76% of conventional jig-based TKA patients. Some of these releases may be necessary, because the surgeons must balance the gaps for possible unknown bone preparation mistakes, but they could be avoided by correct bone cutting strictly following the preoperative plans in RA-TKA [4–8].

### Periarticular soft tissue protection

RA-TKA could utilize haptic feedback boundaries to limit the action of the saw blade to the confines of the preoperative surgical plans for bone resections. Cadaveric studies have shown that RA-TKA is associated with reduced iatrogenic soft tissue envelope injury, such as ligament injury, tibial subluxation and patella eversion, compared to conventional jig-based TKA [9]. The Macroscopic Soft Tissue Injury (MASTI) score has been used to evaluate different zones of periarticular soft tissue, and use of RA-TKA is related to reduced bone and soft tissue injury [10]. Hence, this could be why several studies have reported that RA-TKA is associated with decreased postoperative pain, enhanced early functional rehabilitation and decreased time to hospital discharge [9].

### Avoidance of medullary canal invasion

Jig-based conventional TKA requires the assistance of an intramedullary referencing guide pole for bone resection. One certain benefit of RA-TKA, which can decrease surgical trauma, is the avoidance of femoral medullary canal invasion. Most authors have reported that intraoperative and postoperative blood loss in RA-TKA is always significantly less [11]. The invaded femoral canal could bleed severely during surgery and be responsible for undetectable blood loss after surgery.

In addition, using an intramedullary reference guide pole might also increase the risk of thromboembolic events and cardiorespiratory complications [6,7]. The medullary approach can also influence the overall physical situation by causing microthrombotic embolism or fat infarction in pulmonary arterial terminals [12], although this is temporary and sometimes undetectable. This fact might be why robot-assisted medullary-canal-spared TKA is better than traditional TKA for transient reduction in the early postoperative inflammatory response. It can be tested by related blood tests, which could be used as indicators of postoperative trauma [13].

### Better early functional outcomes

RA-TKA can improve preservation of the soft envelope secondary to reduced intentional soft tissue release and can decrease iatrogenic periarticular soft tissue injury. This process could help to limit the local inflammatory response, decrease pain and reduce postoperative swelling, leading to decreased analgesia requirements, a shorter time for straight leg raise, increased knee flexion at discharge and a reduced need for inpatient physiotherapy [14]. Additionally, RA-TKA can be related to a shorter median time to hospital discharge (77 h compared with 105 h), better patient satisfaction and physical function scores [15] and greater improvements in walking and standing [16].

## Factors that can increase surgical trauma of RA-TKA

### Increased time spent in the operating room

Robot-assisted surgery can increase the surgical time due to robotic arm preparation and registration processes. Khlopas *et al*. concluded that RA-TKA could achieve the same time-consumption level as conventional TKA after the first 15 cases on average [17]. While the common belief is that RA-TKA could eliminate the difference in time spent in the operating room only for lower-volume surgeons who had reached the steep portion of the learning curve, using an image-based robotic system can help to shorten the surgical time with a preoperatively created virtual 3D model.

### Alternative to conventional surgery

Use of the robotic system has sometimes been aborted in the early period of the learning curve because the surgeon was unfamiliar with the system or failed to achieve human–computer interaction during the surgery, which increases the time spent and soft tissue trauma. The rate of intraoperative switching from the robotic to the conventional technique has varied in the literature (1–22%) [18–20]. This phenomenon may be caused by technical issues with robotic devices that require intraoperative conversion to conventional jig-based TKA [21]. This intelligence-aid procedure is still under development, and any surgeon who decides to use it should be both good at robotic surgery and fully qualified to perform traditional surgery.

### Longer incision

The soft-tissue trauma caused by incision and exposure is also longer for robotic surgery. Infrared ray navigation-based TKA robots require two additional 2-cm incisions to fix registration pins to enable optical motion-capture tracking on the femur and tibia. To avoid impingement of soft tissue with terminal pieces of the robot arm, the incision for exploring the knee joint on average exceeds the traditional incision. This is especially true when using an autonomous robot; the surgeon must expose the bone structure for the robotic arm, and the incision is always much larger. With regard to the issue of trauma, the performance of early-stage TKA robots was not satisfactory. Fully active robotic TKA systems have been reported to cause unexpected periarticular soft tissue injury. Newly developed robotic systems have improved registration efficiency, for time consideration, and use electromagnetic trackers to avoid additional incisions for trackers.

### Complication rate

In addition to all of the possible factors mentioned above that might be directly related to surgical trauma, a higher complication rate is another factor that must be considered [4,22]. The prolonged soft tissue exposure time in the operating room increases the risk of contamination by microorganisms, which could lead to a higher infection rate. Infrared sensors reduce the intraoperative working space, and additional instruments and surgical trays can cause instrument crowding and prolonged working times, further increasing the risk of contamination. The prolonged tourniquet time consequently led to increased ischemia–reperfusion injury, severity of knee swelling and lower extremity oedema [23]. In addition, complications such as pin-tract infection and pin-site fracture should not be ignored [24].

## Debated benefits of RA-TKA in decreasing trauma

The use of robotics in TKA is growing at an exponential rate [25]. Despite the improved accuracy and reproducibility of robotic-assisted TKA, consistent clinical benefits have yet to be determined. No differences in medium- to long-term functional outcomes have been found between conventional jig-based TKA and robotic surgeries assisted by other types of robots [9]. The longest clinical results for RA-TKA averaged 13 years in follow-up studies of the first-generation autonomous robot, Robodoc [26]. No significant differences were found in the functional score, loosening rate or prosthetic survivor rate. A recently published study demonstrated no real benefits of this device [27].

There are factors that favour and oppose decreasing the surgical time for RA-TKA. As the authors of the commented article concluded, robot-assisted bone preparation and avoidance of unnecessary soft tissue release could decrease the surgical time that would otherwise be prolonged [1]. This conclusion partially echoes the literature reporting no difference in postoperative trauma, although increased registration time and length of incision could result in surgical trauma [28–30]. Although some short-term follow-ups have shown good clinical results for RA-TKA, the final conclusion regarding whether robotic surgery will decrease surgical trauma remains unknown [31]. The reasons might include modern TKA having been designed so successfully that it renders measurable improvements in the clinical outcomes of robotic assistance difficult to test.

## Conclusions

Despite all of the desired advantages of robot-assisted surgery, debates about its expanding usage are ongoing. Surgical trauma is one of the important issues influencing the decision to use robots. There are factors favouring RA-TKA, such as periarticular soft tissue protection, improved early functional performance, avoidance of medullary canal invasion and unnecessary soft tissue release. However, there are other factors related to RA-TKA that require further improvement, such as increased operating room time, complications, incisions and learning-curve mistakes. Further high-quality multicentre studies with longer-term follow-ups and larger sample sizes are needed to answer more questions concerning the perioperative trauma due to robotic surgery.

## Abbreviations

RA-TKA: robot-assisted total knee arthroplasty.

## Data Availability

All data and materials of the present study were in full compliance with the journal’s policy.
